# Smart osteoclasts targeted nanomedicine based on amorphous CaCO_3_ for effective osteoporosis reversal

**DOI:** 10.1186/s12951-024-02412-9

**Published:** 2024-04-05

**Authors:** Biao Yu, Qianmin Gao, Shihao Sheng, Fengjin Zhou, Zhen Geng, Yan Wei, Hao Zhang, Yan Hu, Sicheng Wang, Jianping Huang, Mengmeng Li, Jiacan Su

**Affiliations:** 1 Institute of Translational Medicine, Shanghai University, Shanghai, 200444 China; 2 Musculoskeletal Organoid Research Center, Shanghai University, Shanghai, 200444 China; 3 School of Medicine, Shanghai University, Shanghai, 200444 China; 4 Second Affiliated Hospital, Shanghai University, Wenzhou, 325000 China; 5 Department of Orthopedics Trauma, Shanghai Changhai Hospital, Naval Medical University, Shanghai, 200433 China; 6 Department of Orthopedics, Honghui Hospital, Xi’an Jiao Tong University, Xi’an, 710000 China; 7 Department of Orthopedics, Xinhua Hospital, Shanghai Jiao Tong University School of Medicine, Shanghai, 200092 China; 8 Department of Orthopedics, Shanghai Zhongye Hospital, Shanghai, 200941 China; 9 Department of Neurology, Wenzhou Central Hospital, Wenzhou, 325000 China

**Keywords:** pH responsiveness, Oroxylin A, Osteoporosis, Acidic microenvironment, Synergistic therapy

## Abstract

**Background:**

Osteoporosis is characterized by an imbalance in bone homeostasis, resulting in the excessive dissolution of bone minerals due to the acidified microenvironment mediated by overactive osteoclasts. Oroxylin A (ORO), a natural flavonoid, has shown potential in reversing osteoporosis by inhibiting osteoclast-mediated bone resorption. The limited water solubility and lack of targeting specificity hinder the effective accumulation of Oroxylin A within the pathological environment of osteoporosis.

**Results:**

Osteoclasts’ microenvironment-responsive nanoparticles are prepared by incorporating Oroxylin A with amorphous calcium carbonate (ACC) and coated with glutamic acid hexapeptide-modified phospholipids, aiming at reinforcing the drug delivery efficiency as well as therapeutic effect. The obtained smart nanoparticles, coined as OAPLG, could instantly neutralize acid and release Oroxylin A in the extracellular microenvironment of osteoclasts. The combination of Oroxylin A and ACC synergistically inhibits osteoclast formation and activity, leading to a significant reversal of systemic bone loss in the ovariectomized mice model.

**Conclusion:**

The work highlights an intelligent nanoplatform based on ACC for spatiotemporally controlled release of lipophilic drugs, and illustrates prominent therapeutic promise against osteoporosis.

**Supplementary Information:**

The online version contains supplementary material available at 10.1186/s12951-024-02412-9.

## Introduction

Osteoporosis is a typical age-related disorder distinguished by bone mass loss, micro-architecture deterioration, and subsequent increased bone susceptibility and fragility to fracture [1–3]. Preventing osteoporosis is an effective strategy for mitigating age-related bone fractures, particularly in postmenopausal women [4–8]. Given the global population suffering from osteoporosis is estimated at over 200 million, its medical and socio-economic impacts are expected to escalate in today’s aging world [9–11]. From a pathological perspective, osteoporosis occurs as a result of an imbalance between bone catabolism and anabolism [11–13]. Osteoclasts, the specialized cells responsible for bone resorption, play a pivotal role in age-related bone mineral dissolution through the excessive secretion of acid and enzymes, resulting in irreversible loss of bone mass [14–16].

A variety of anti-resorptive drugs have been used clinically for primary bone loss by suppressing osteoclast activity, including selective estrogen receptor modulators, bisphosphonates and denosumab [17–19]. However, following the onset of bone degradation, most of these therapeutic interventions prove inadequate in fully remedying the bone damage induced by osteoclast hyperactivation. Prolonged use of these medications may result in significant adverse effects, limiting their efficacy and availability. For instance, bisphosphonates have been associated with rare cases of atypical femoral fractures and jaw osteonecrosis [20–22]. Estrogen therapy may increase the risk of certain types of breast cancer and vascular events [23, 24].

In ancient Asian countries, traditional herbs have been used for centuries to enhance bone health and treat various musculoskeletal disorders [25–27]. The widely used herb, *Scutellaria baicalensis*, in traditional Chinese medicine has shown a variety of pharmacological activities, particularly in improving bone mineral density and enhancing the microstructure of bone [28–30]. Oroxylin A (ORO) is one of the most biologically active compounds extracted from *Scutellaria baicalensis* and is considered a promising candidate for treating osteoporosis and fracture healing [31–33]. We previously demonstrated that ORO inhibits RANKL-induced osteoclastogenesis. Furthermore, in vivo studies showed that intraperitoneal injection of ORO maintained bone mass in ovariectomy-induced osteoporosis mice and accelerated bone fracture healing [34]. However, it’s worth noting that the natural flavonoid ORO suffers from a lack of targeting specificity and poor water solubility, which result in off-target effects and low bioavailability for osteoporosis therapy [35–37]. The design strategy of nanocarriers with precise and smart properties could achieve controlled drug delivery in a temporally and spatially regulated manner, thus improving its effective accumulation within the pathological microenvironment [38, 39]. During osteoporotic diseases, osteoclasts exert excessive acid etching in the resorption zone, and this acidified niche (pH ∼ 4.5 ± 0.3) offers the potential for responsive release of bone-targeted drugs [40–42]. Furthermore, neutralization of the acidified osteoclast microenvironment and supplement of calcium is proven to promote osteoclast apoptosis and alleviate osteoporosis [43–46]. To achieve this, phospholipid-coated amorphous calcium carbonate (ACC) is employed as acid-responsive nanocarriers for the delivery of water-soluble drugs with high loading capacity and rapid drug release [47–49]. However, when it comes to lipophilic flavonoids typically loaded into the phospholipid (PL) bilayer, there is a lack of pH-responsive sensitivity.

**Scheme 1:**
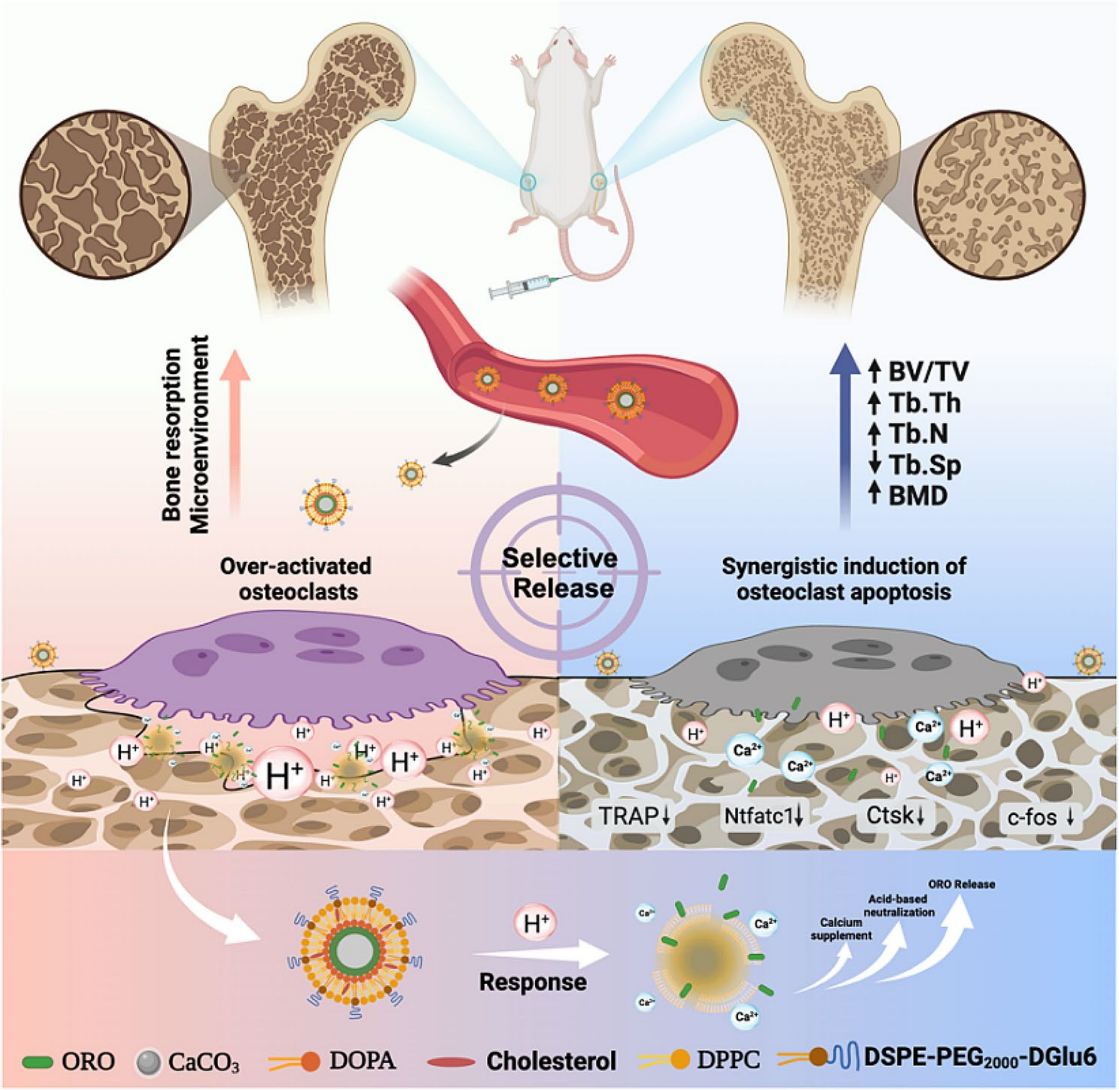
Schematic illustration of OCs-targeted OAPLG for osteoporosis therapy

Herein, ORO was immobilized onto the surface of ACC and subsequently modified with PL and a bone-targeting peptide, D-glutamic acid hexapeptide (DGlu6), on the surface, able to isolate the ORO/ACC from an external aqueous media and trigger ORO release in response to acidified microenvironment. This intelligent nano-platform ORO/ACC/PL/Glu6, termed OAPLG, endowed ORO with bone-targeted and pH-responsive drug release properties. We hypothesize that OAPLG nanoparticles can spontaneously create a nanolayer on the surface of the bone. Once osteoclasts secrete excess H^+^ ions, OAPLG is activated due to pH sensitivity, which leads to the release of ORO, neutralizes the acidic environment, and replenishes lost calcium. Combining ORO, an osteoclast inhibitor, with calcium carbonate, an acid neutralizer, is expected to synergistically suppress osteoclast activity and improve osteoporosis outcomes. In this study, we confirmed the specific targeting ability of OAPLG to bone and the responsive release profile to the acidity mediated by osteoclasts, which presented superior inhibition of osteoclast activity in vitro as well as significant therapeutic efficacy of osteoporosis in vivo. Thus, the OAPLG nanoplatform not only greatly enhanced drug bioavailability by releasing lipophilic flavonoids in response to the acidic niche of osteoclasts, but also reversed the pathological microenvironment, thus synergistically inhibiting bone loss (Scheme 1).

## Materials and methods

### Materials

Ammonium Carbonate ((NH_4_)_2_CO_3_, 99%), Calcium Chloride (CaCl_2_, 99%), and polyvinyl pyrrolidone (PVP, K30) were purchased from Shanghai Tian Scientific Co., Ltd. Oroxylin A (ORO) was purchased from Shanghai Standard Technology Co., Ltd.

DPPC and cholesterol were purchased from AVT (Shanghai) Pharmaceutical Tech Co., Ltd. Receptor activator of nuclear factor-κB ligand (RANKL, 95%) was purchased from R&D systems Co., Ltd. Macrophage colony-stimulating factor (M-CSF, 98%) were purchased from PeproTech Co., Ltd. DSPE-PEG_2000_-DGlu6 was customized by ChinaPeptides Co., Ltd. CTX-I ELISA Kit and PINP ELISA Kit were purchased from Shanghai Lengton Bioscience Co., Ltd. All primers were synthesized by Sangon Biotech Co., Ltd.

### Synthesis of DSPE-PEG_2000_-DGlu6

DSPE-PEG_2000_-DGlu6 was obtained through a conjugation reaction between DSPE-PEG_2000_-Mal and DCys-DGlu6. In brief, DCys-DGlu6 and DSPE-PEG_2000_-Mal (4:1, mol/mol) were dissolved in a solution of ACN/H_2_O (2:1, v/v). The mixture was stirred, and then 0.2 M PBS and 6 M HCl solution (pH = 7.0) were added. Nitrogen gas was used to purge the mixture, replacing oxygen, and the reaction was conducted for two hours in a nitrogen environment at room temperature. The remaining unreacted DCys-DGlu6 peptide was subsequently removed by dialysis (MWCO, 1000 Da). The resulting product, DSPE-PEG_2000_-DGlu6, was then freeze-dried to obtain the final product.

### Preparation of OAPLG

ACC was prepared using a steam diffusion method [48]. Initially, 200 mg of CaCl_2_ was placed in a 200 ml round-bottom flask. After completely dissolving the CaCl_2_ in 300 μl of distilled water, 100 mL of anhydrous ethanol was added. The flask was tightly sealed with a film and punctured with a needle to create small holes. The round-bottomed flask was placed in a desiccator along with glass vials containing (NH_4_)_2_CO_3_ and reacted at 37 °C for 24 h. Afterward, the product was purified by centrifugation (8000 rpm,10 min), washed several times with anhydrous ethanol, and then dispersed in anhydrous ethanol for storage at 4 °C. To prepare the OCA nano-core, an ORO ethanol solution (24 mg, 1 mL) was added to a CaCO_3_ ethanol solution (6 mg, 2 mL) containing PVP molecules (20 mg). The mixture was agitated at 25 °C for 4 h, and the resulting drug-coated CaCO_3_, referred to as the OCA nano-core, was collected.

The ethanol solution of the OCA nano-core (20 mg, 5 mL) was mixed with a chloroform solution of DOPA (2 mg, 1 mL), which was purchased from AVT (Shanghai) Pharmaceutical Tech Co. The mixture was then subjected to 40 min of water bath sonication. The DOPA-coated nanoparticles were then purified through centrifugation. The resulting particles were re-suspended in a chloroform solution (6 mL) containing cholesterol (2 mg) (AVT (Shanghai) Pharmaceutical Tech Co.), DPPC (4 mg), and DSPE-PEG_2000_-DGlu6(8 mg) and then stirred overnight. The chloroform was removed using a rotary evaporator, and the particles were rehydrated with PBS (2 mL) under the influence of ultrasonic waves. The polyethylene glycol-coated nanoparticles were collected and purified through centrifugation (8000 rpm, 10 min) before being stored at 4 °C for further experimentation.

### Drug loading content

The drug loading of OAPLG was determined using UV-Vis spectrophotometry. The measurement was performed at a wavelength of 272 nm. To disrupt the OAPLG dispersion, a 1:1 mixture of 1 M HCl and ethanol was added, effectively breaking down the OAPLG particles. The free ORO was obtained by high-speed centrifugation (20,000 rpm, 15 min). The drug loading percentage (DL%) of ORO was calculated as follows:

$${\rm{DL\% }}\,{\rm{ = }}\,\left( {{{\rm{W}}_{\rm{A}}}{\rm{/}}\,\left( {{{\rm{W}}_{\rm{A}}}{\rm{ + }}\,{\rm{W}}} \right)} \right)\,{\rm{ \times }}\,{\rm{100\% }}{\rm{.}}$$

Here, W_A_ represents the weight of the free drug in the supernatant, while W represents the total weight of the system.

### Drug release

To investigate drug release, 2 ml of OAPLG solution containing a consistent ORO concentration of 200 μg was placed in a dialysis bag with a molecular weight cut-off of 7,000 Da, which was then immersed in a centrifuge tube containing 25 ml of phosphate buffer solution of different pH (pH 4.5/6.5/7.4). Samples of 0.5 ml were collected at different time points under constant temperature conditions at 37 °C and a rotational speed of 100 rpm. After each sampling, an equal volume of the release medium at the same temperature was promptly replenished. The concentration of ORO was quantified with a UV-Vis spectrophotometer.

### pH responsiveness on bone surface

The DiD-labeled OAPLG was added to a 96-well plate containing bone slices, incubated in complete medium for 12 h at 37 °C, and then the medium was removed; after three washes with physiological saline, the samples were replenished with complete α-MEM medium. At 1, 3, and 7 days, the DiD fluorescence signal was measured. The co-incubation was then continued by replacing the medium with an acidic buffer solution (pH 4.5). A series of images were taken over a period of 5 min to observe changes in DiD fluorescence.

### Extraction and cultivation of BMMs

BMMs were extracted from the femur and tibia of C57BL/6 mice (4 weeks, female). The mice were euthanized using 3% sodium pentobarbital, and femurs and tibiae were obtained from the hind limbs in a sterile environment after 10 min of immersion in 75% ethanol. The ends of the long bones were cut to expose the marrow cavity. Subsequently, the femurs and tibiae were washed 2–3 times in a cell culture dish containing PBS and transferred to another cell culture dish containing complete growth medium. The bone ends of the femur and tibia were opened using ophthalmic scissors, and 1 mL of complete growth medium was aspirated with a syringe to flush the bone marrow cells from one end of the bones into a sterile 50 mL centrifuge tube. This process was repeated multiple times until the bones turned white. The erythrocytes were lysed with erythrocyte lysis buffer, and the cell pellet was collected by centrifugation, resuspended in α-MEM cell culture medium, and filtered through a 200-mesh sieve. The cells were subsequently suspended in 6 mL of α-MEM growth medium containing 25 ng/mL macrophage colony-stimulating factor (M-CSF; R&D Systems) for 3 days. BMMs were the only cells able to adhere and survive under M-CSF stimulation, so the culture flask’s adherent cells at the base were recognized as BMMs. When the adherent cells reached approximately 90% confluence, they were harvested by digesting with trypsin (Gibco) for 15 min and used for subsequent in vitro experiments.

### Cell toxicity assay

BMMs were added to 96-well cell culture plates containing complete α-MEM medium at a density of 1 × 10^4^ cells/well and cultured for 24 h to ensure complete spreading. Subsequently, different concentrations of ORO and the nanocarrier were introduced to the cells, and they were cultured for either 24 or 72 h. Then, 10 μl of CCK-8 was added to each well and incubated at a constant temperature for a period of time to measure the absorbance of the plate using a multifunctional microplate reader. The absorbance value for the blank medium group was set to 100%.

### TRAP staining

BMMs were cultured in 96-well plates (8000 cells/well) in complete growth medium containing both 25 ng/mL M-CSF and 50 ng/mL RANKL. To promote osteoclast maturation, the culture medium was changed every two days. After 5 days of induction culture, cells were fixed and stained using a TRAP staining kit (Sigma, St. Louis, USA). Mature osteoclasts were identified as cells with three or more nuclei, and Image J software was utilized for further image analysis.

### Bone resorption assay

BMMs (1 × 10^4^ cells/well) were seeded onto a 96-well plate containing small bovine bone slices and induced for 10 days as previously described. Following that, osteoclasts were removed using 5% sodium hypochlorite, and the bone slices were subsequently stained with 1% toluidine blue for 2 min. An optical microscope was used to examine the absorption area of the bone slices. Image J software was utilized for further image analysis.

### Fluorescent staining of F-actin rings

As described above, osteoclasts were induced in different treatment groups for 5 days. The cells were fixed and permeabilized with Triton-X100. To visualize F-actin within the cells, Fluorescein isothiocyanate (FITC)-labeled phalloidin staining was carried out. Additionally, the nuclei were stained with DAPI. Confocal microscopy images of F-actin rings were acquired (FITC Ex/Em = 488/525 nm, DAPI Ex/Em = 340/488 nm). Image J software was utilized for further image analysis.

### Real-time quantitative PCR

BMMs (4 × 10^5^ cells/well) were seeded evenly in a 6-well plate with α-MEM complete medium containing RANKL. The cells were treated with ORO, APLG, OAPL, and OAPLG, respectively, and blank and positive controls were included. After 5 days of incubation with medium changes every two days, total RNA was extracted from the cells utilizing RNAiso Plus (TaKaRa, Japan). Reverse transcription was performed using PrimeScript™ RT Master Mix (TaKaRa, Japan), and finally, qPCR was performed using MonAmp™ SYBR® Green qPCR Mix (Monad, China). The primers used were those reported in previous studies and their sequences are presented in Supplementary Table S1 [44].

### Western blotting

The protein levels of NFATc1 (sc-7294, Santa), c-fos (2250, CTST), Ctsk (4980, CTST), and GAPDH (ab181602, Abcam) were detected using Western blotting. To extract the proteins, cells were lysis by adding RIPA Lysis Buffer (Beyotime, China). SDS-PAGE was used to separate the extracted proteins. The proteins were transferred onto a PVDF membrane after electrophoresis. Skim milk was then used to block the membrane. The membrane is washed and incubated with primary antibody overnight with gentle shaking, then incubated with secondary antibody for 2 h at room temperature. An enhanced chemiluminescence substrate was used to detect the antibodies’ chemiluminescent signal. ImageQuant LAS 4000 system (GE Healthcare, Silverwater, Australia) was used to capture images, which were then analyzed with ImageJ software.

### In vitro bone targeting of OAPLG

To examine the binding capacity of bone matrix and bone-targeting delivery systems in vitro, a bone slice adsorption experiment was conducted. In simple terms, DiD-labeled OAPL and OAPLG were added to a 96-well plate containing bone slices. The plate was gently oscillated at room temperature to facilitate the adsorption of DiD-labeled OAPL and OAPLG onto the bone slices. The adsorption of DiD-labeled OAPL and OAPLG was quantified by measuring the difference in fluorescence intensity between the beginning stock solution and the supernatant after adsorption, using the following formula:

$${\rm{Absorption}}\,{\rm{affinity}}\,{\rm{ = }}\,\left( {{{\rm{I}}_{\rm{0}}}{\rm{ - }}{{\rm{I}}_{\rm{a}}}} \right)\,{\rm{/}}\,{{\rm{I}}_{\rm{0}}}$$

In this equation, I_0_ refers to the initial fluorescence intensity of the stock solution, while I_a_ refers to the fluorescence intensity of the supernatant after 1 h of incubation.

### In vivo bone targeting of OAPLG

All experiments related to the animals involved in this study were conducted in strict accordance with the Guidelines for the Care and Use of Laboratory Animals and approved by the Animal Care and Use Committee of Shanghai University. C57BL/6 mice were used as the experimental subjects to evaluate the bone-targeting ability of OAPLG. The mice were given an intravenous injection of either 100 μL of PBS or a solution containing nanoparticles labeled with DiD, with or without Dlu6 surface modification (DiD concentration of 20 μg/mL). At 4 and 8 h after nanoparticle administration, the mice were euthanized under anesthesia. Tissue samples were collected for in vivo analysis to investigate the biodistribution of the DiD-labeled nanoparticles.

### Establishment and treatment of osteoporosis mouse model(OVX)

A total of 30 female C57BL/6 mice (6 weeks) were purchased from Changzhou Cavens Experimental Animals Co., Ltd. (Changzhou, China). After a two-week acclimation period, 25 mice were randomly selected to undergo ovariectomy surgery. Following anesthesia, the dorsal fur was shaved, and the mice were placed in a supine position. The dorsal skin was disinfected, and a 1 cm longitudinal incision was made along the midline. Careful dissection was performed to expose the ovaries by removing the surrounding adipose tissue. After ligating the fallopian tubes, both ovaries were completely excised, and the incision was closed and disinfected with iodine. The remaining 5 mice underwent the sham surgery group, where the same procedure was performed, but the ovaries were preserved. After one month post-surgery, 25 OVX mice were randomly assigned to 5 groups. Finally, a total of 6 groups were established: sham surgery group, OVX group, OVX + ORO group, OVX + APLG group, OVX + OAPL group, and OVX + OAPLG group. ORO, APLG, OAPL, and OAPLG solutions were administered every two weeks at a controlled drug concentration of 0.1 mg/kg. The sham surgery group and OVX group received intravenous injections of normal saline. The treatment duration was 2 months. Upon completing the experiment, peripheral blood samples were obtained through enucleation under anesthesia. Subsequently, the mice were euthanized to retrieve bilateral femurs as well as major organs. All collected tissues were subsequently fixed in 4% PFA for further experiments. Micro-CT analysis (Bruker micro CT, Belgium) was performed to assess alterations in femoral trabecular bone. The SkyScan-1176 system was used with a voxel size of 13 μm, 90 kV, 278 μA, exposure time of 230 ms, 0.5 mm aluminum filter, and a rotation step of 180°. Three-dimensional reconstruction and image visualization were conducted using NR Economy software version 1.6. After three-dimensional reconstruction, bone analysis was performed using CT software version 1.13. The assessed parameters encompassed BMD, BV/TV, Tb. Th, Tb. N, and Tb. Sp, serving as indicators for evaluating bone repair conditions.

### Tissue histological analysis

The femur samples from the mice were placed in a 10% EDTA solution for the purpose of decalcification, with regular solution changes occurring every 3 days. After decalcification, the samples underwent gradient ethanol dehydration and were then made transparent by exposure to xylene solution. Later, the samples were immersed in liquid paraffin and left to solidify before obtaining 4 μm thick sections along the longitudinal axis. These sections were subjected to staining using a combination of H&E reagents, Masson’s reagent, and a TRAP staining kit. The major organs of the mice were fixed and H&E stained to further evaluate the safety of various drug treatments.

## Results and discussion

### Preparation and characterization of OAPLG nanoparticles

**Fig. 1:**
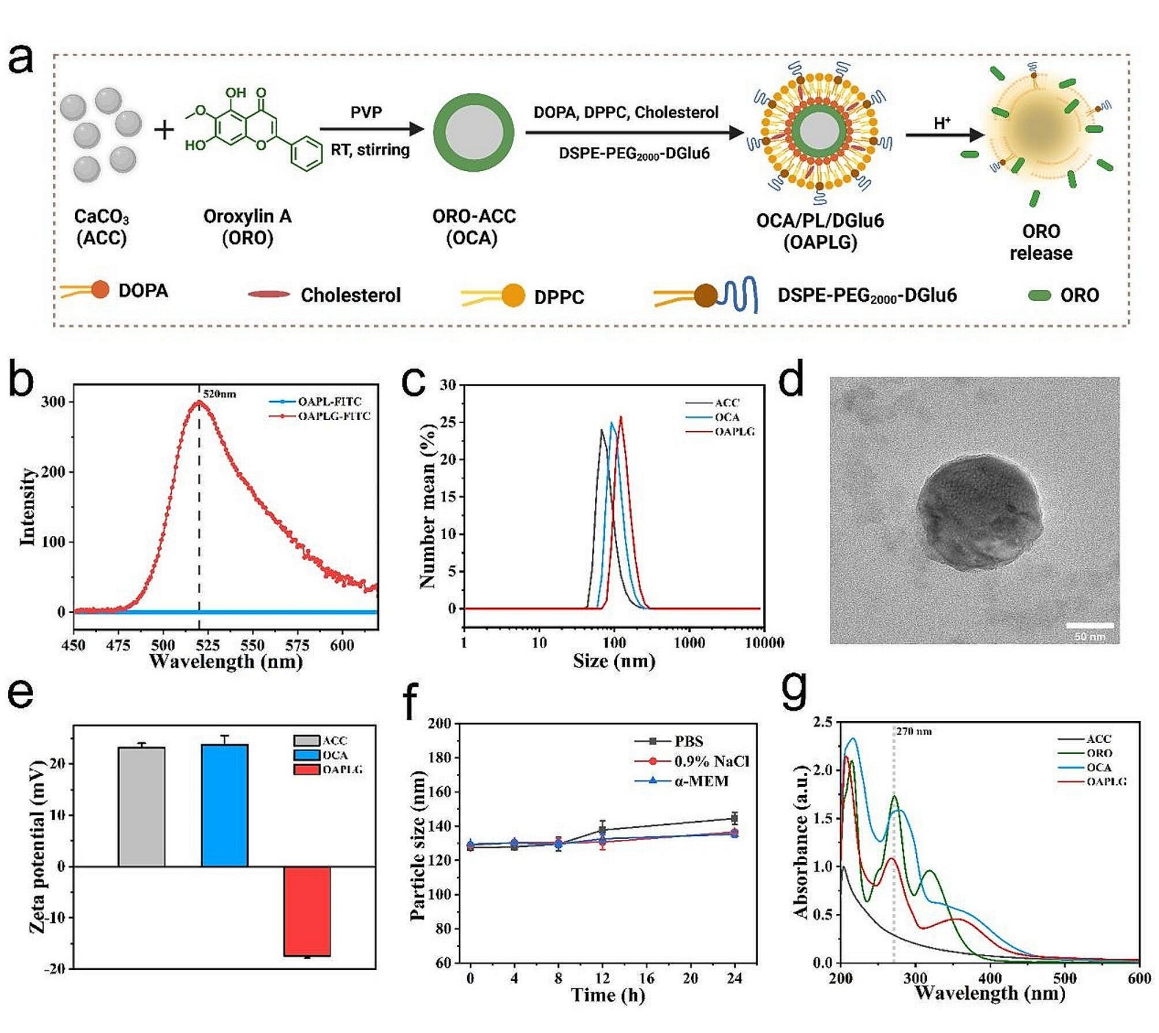
Characteristics of the nanoplatform OAPLG. (**a**) Preparation of OAPLG loaded with ORO. (**b**) Fluorescence intensity of OAPL and OAPLG nanoparticles with FITC-modified DSPE-PEG_2000_-DGlu6 as obtained on a fluorescence spectrophotometer. (**c**) The particle size of ACC, OCA and OAPLG measured by DLS. (**d**) TEM image of a representative OAPLG. Scale bar = 50 nm. (**e**) Surface Zeta potentials of ACC, OCA, OAPLG. (**f**) Detection of OAPLG particle size over time in different buffers. (**g**) UV-vis absorption curves of ORO, ACC, OCA, and OAPLG

To construct the OAPLG core, which enables simultaneous osteoclast activity inhibition and neutralization of produced acidity, amorphous CaCO_3_ (ACC), obtained via the gas diffusion method, was coated with water-insoluble ORO via the aid of stabilizer PVP by a one-pot approach (Fig. 1a). ORO-coated ACC, abbreviated to OCA, nanoparticles were obtained by mixing ACC with ORO at a mass feeding ratio of 1:4. Next, the obtained OCA core was further surface modified with a lipid layer and D-glutamic acid hexapeptide (DGlu6). To link the bone-targeting peptide DGlu6 to the formed lipid layer, the amino terminus of DGlu6 was covalently linked to DCys, thereby introducing a free thiol group (-SH) and yielding DCys-DGlu6. Subsequently, DCys-DGlu6 underwent thiol-maleimide addition reaction with DSPE-PEG_2000_-Mal, enabling the synthesis of DSPE-PEG_2000_-DGlu6. As demonstrated in the MALDI-TOF-MS spectrum, the MW of DSPE-PEG_2000_-Mal measures nearly 2900 (Fig. S1a). Subsequent cross-linking of DCys-DGlu6 resulted in an increase in the product’s MW to approximately 3800 (Fig. S1b). This observed MW difference of approximately 900 aligns with the theoretical MW of DCys-DGlu6 (Fig. S1c). Furthermore, OCA core sequentially coated with DOPA, DPPC, cholesterol, and DSPE-PEG_2000_-DGlu6 at a mass ratio of 1:2:1:4 by forming phospholipid lipid layers (PL) on their surface via two-step self-assembly method [50], to create hybrid nanoparticles while retaining the bone-targeting nature of ORO/ACC/PL/DGlu6 (abbreviated to OAPLG). Meanwhile, the hybrid nanoparticles were fabricated without bone-targeting peptides as control (abbreviated to OAPL). To ascertain whether DGlu6 was successfully inserted within the OAPLG lipid layer, fluorescein FITC was used to conjugate the amino terminus of DSPE-PEG_2000_-DGlu6. Fluorescence spectrophotometry revealed a substantial increase in intensity at 520 nm for FITC-modified OAPLG, indicating the colocalization of bone-targeting peptides with the lipid membrane on OAPLG (Fig. 1b).

The ACC core within OAPLG nanoparticles maintained its amorphous state, as confirmed by the analysis of selected area electron diffraction (SAED) (Fig. S2a), which was expected to enable instant drug release [51]. The size of the modified hybrid nanoparticles (OAPLG) was determined to be approximately 140 nm, slightly larger than that of the OCA and ACC nanoparticles, as shown in Fig. 1c and S2a. TEM images revealed a thin corona with a discernible membrane-core structure, depicted in Fig. 1d. Zeta potential measurements indicated that ACC and OCA nanoparticles exhibited a positive charge due to the presence of additional Ca^2+^ on their surfaces [52]. In contrast, the zeta potential of OAPLG was negative (-17.43 ± 0.42 mV), attributed to the surface anchoring of PL and DSPE-PEG_2000_-DGlu6 (Fig. 1e). The change in surface zeta potential from positive to negative provided compelling evidence of successful OAPLG modification with a membrane. This modification is expected to isolate the OCA core from the aqueous environment, preventing ACC hydration and nanoparticle aggregation in aqueous environments [53]. Structural stability tests conducted in PBS, 0.9% NaCl, and α-MEM medium demonstrated that OAPLG nanoparticles remained significantly stable, thus suitable for following chemical and biological experiments (Fig. 1f). Additionally, using the mass extinction coefficient of ORO molecules at 270 nm, the ORO content in OAPLG was calculated to be 41.7%, which demonstrates the nanoplatform’s exceptional high-load performance (Fig. 1g and S2c). These findings collectively confirm the successful synthesis of OAPLG nanoparticles.

### pH responsiveness and bone targeting properties of OAPLG

**Fig. 2:**
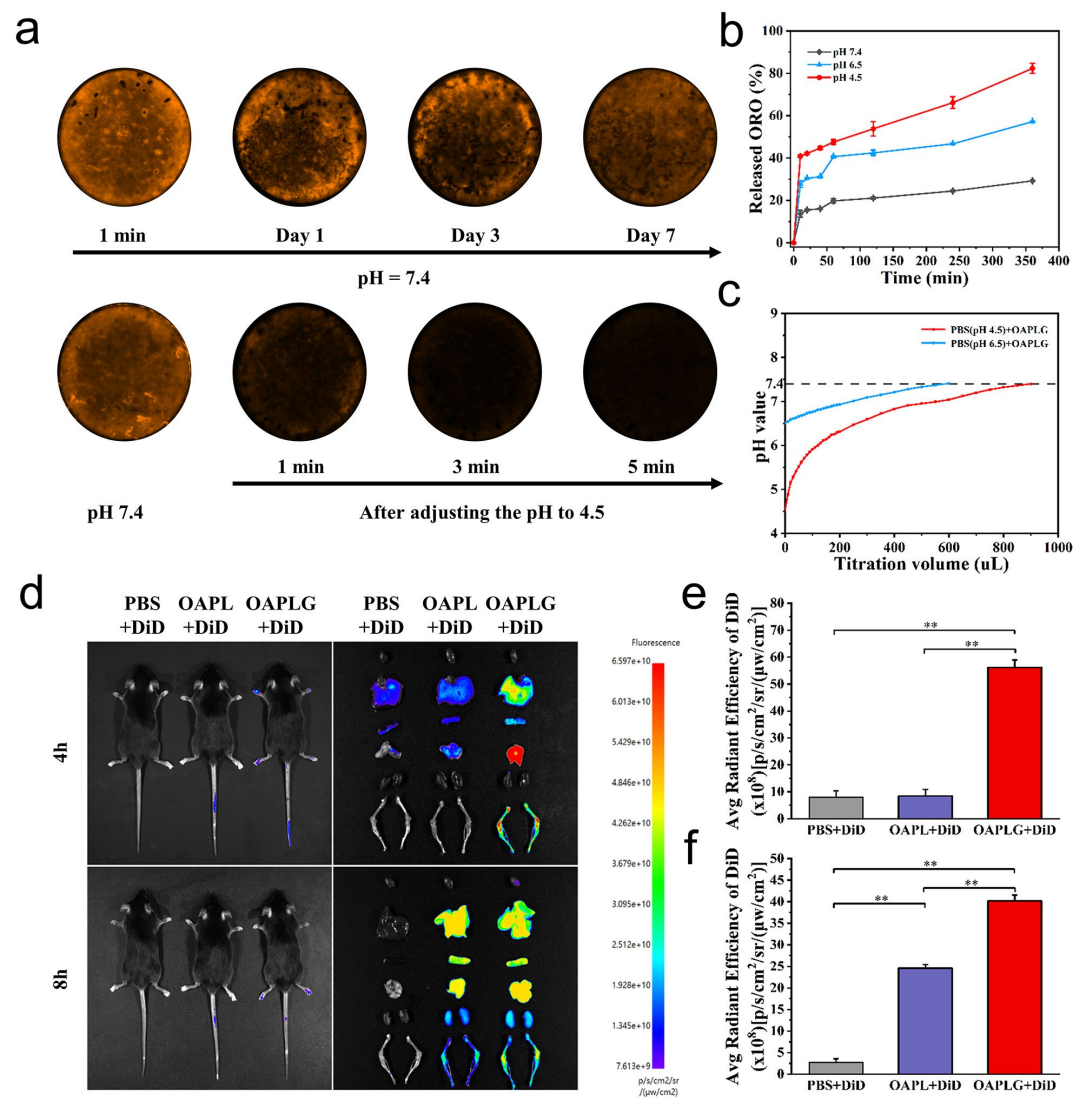
pH responsiveness and bone targeting properties of the nanoplatform OAPLG. (**a**) Following incubation with DiD-labeled OAPLG at pH 7.4, the fluorescence signal of DiD on the surface of the bone slice was assessed at time intervals of 1 min, 1 day, 3 days, and 7 days. Subsequently, the pH was rapidly adjusted to 4.5, and the DiD fluorescence signal was measured at intervals of 1 min, 3 min, and 5 min. (**b**) Drug release curves of OAPLG in different pH buffer solutions over time. (**c**) Detection of pH value in OAPLG titration of different pH buffer solutions. (**d**) Fluorescence signals in live as well as isolated organs and femur and tibia of mice 4 and 8 h after intravenous injection of DiD-labeled OAPLG.(**e**) Quantification of fluorescence signals in femur and tibia 4 h after intravenous injection of DiD-labeled OAPLG. (**f**) Quantification of fluorescence signals in femur and tibia 8 h after intravenous injection of DiD-labeled OAPLG. *n* = 3. *P < 0.05, **P < 0.01, ***P < 0.001

H^+^ penetration into the lipid layer occurs through the creation of transient pores, initiating subsequent reactions with the inner ACC to induce the release of content [44]. Thus, we hypothesize that OAPLG can exhibit pH-responsive behavior. To confirm the OAPLG’s response to acidity on simulated bone surfaces, DiD-labeled OAPLG was co-incubated with the bovine bone slices in the complete culture medium, and the fluorescence signal of DiD/OAPLG on the bone surface was observed. Notably, OAPLG remained stably distributed on the bone slices in the medium with pH of 7.4 for 7 days. But when the pH was dropped to approximately 4.5, similar to the acidity of the local extracellular microenvironment of active OCs, the OAPLG fluorescence signal soon vanished after a short period, indicating its strong pH-responsive nature (Fig. 2a). Subsequently, we examined the ORO release behavior of OAPLG in response to different buffer solutions with pH values of 4.5, 6.5, and 7.4, respectively. ∼82.32% ORO was released from OAPLG after 6 h at pH 4.5, and ∼ 57.16% ORO was released from OAPLG at pH 6.5, demonstrating that OAPLG has sensitivity towards the acid environment (Fig. 2b). Moreover, once incubation of OAPLG in aqueous solutions with varying pH levels, we observed the disintegration of OAPLG in acidic conditions via DLS analysis. At pH values of 7.4, 6.5, and 4.5, OAPLG nanoparticles displayed a noticeable increase in size, suggesting an acid-triggered disassembly process (Fig. S2b). This phenomenon can be attributed to the formation of CO_2_ bubbles and subsequent swelling of the lipid membrane [54]. In addition to ORO release and accumulation around mature osteoclasts, acid neutralization of osteoclasts’ extracellular environment is also believed to accelerate cell apoptosis [44]. Thereby, we further analyzed the acid neutralization and buffering capacity of OAPLG. As illustrated in Fig. 2c, upon the introduction of OAPLG into acidic PBS solutions with pH values of 4.5 and 6.5, the pH of the PBS solutions notably increased, demonstrating OAPLG’s ability to reverse acidity. Furthermore, during titration with 1% HCl, the OAPLG group displayed a clear deceleration in pH decline, indicating its buffering capacity in mitigating extracellular acidification (Fig. S2d).

Next, we evaluated the bone targeting property of OAPLG both in vitro and in vivo. As depicted in Fig. S3, the assay revealed marked differences in the fluorescence intensity of the supernatant between the DiD-labeled OAPL and DiD-labeled OAPLG groups after 1 h of incubation. Approximately 80.6% of OAPLG was found to be adsorbed onto the bone slices, indicating a remarkable enhancement in bone targeting facilitated by DSPE-PEG_2000_-DGlu6, consistent with previous studies [55]. Additionally, mice received intravenous injections of DiD-loaded OAPL and OAPLG solutions to facilitate non-invasive tissue distribution monitoring via the In Vivo Imaging System (IVIS). After caudal intravenous injection, the OAPL group exhibited minimal fluorescence signals within musculoskeletal tissues during the initial 4-hour period. In contrast, the OAPLG group demonstrated a significantly higher fluorescence signal, primarily localized in the femurs, with a 6.6-fold higher intensity compared to the OAPL group (Fig. 2d **and e**). Furthermore, even after an additional 4-hour interval, the OAPLG group still exhibited a notably more substantial increase in fluorescence within the femurs compared to the OAPL group (Fig. 2d **and f**). These findings provide compelling evidence that the DGlu6 modification substantially enhances the targeted accumulation of OAPLG within bone tissue, implying its promising potential for targeted osteoporosis therapy.

### Inhibition of OCs biological activity by OAPLG

**Fig. 3:**
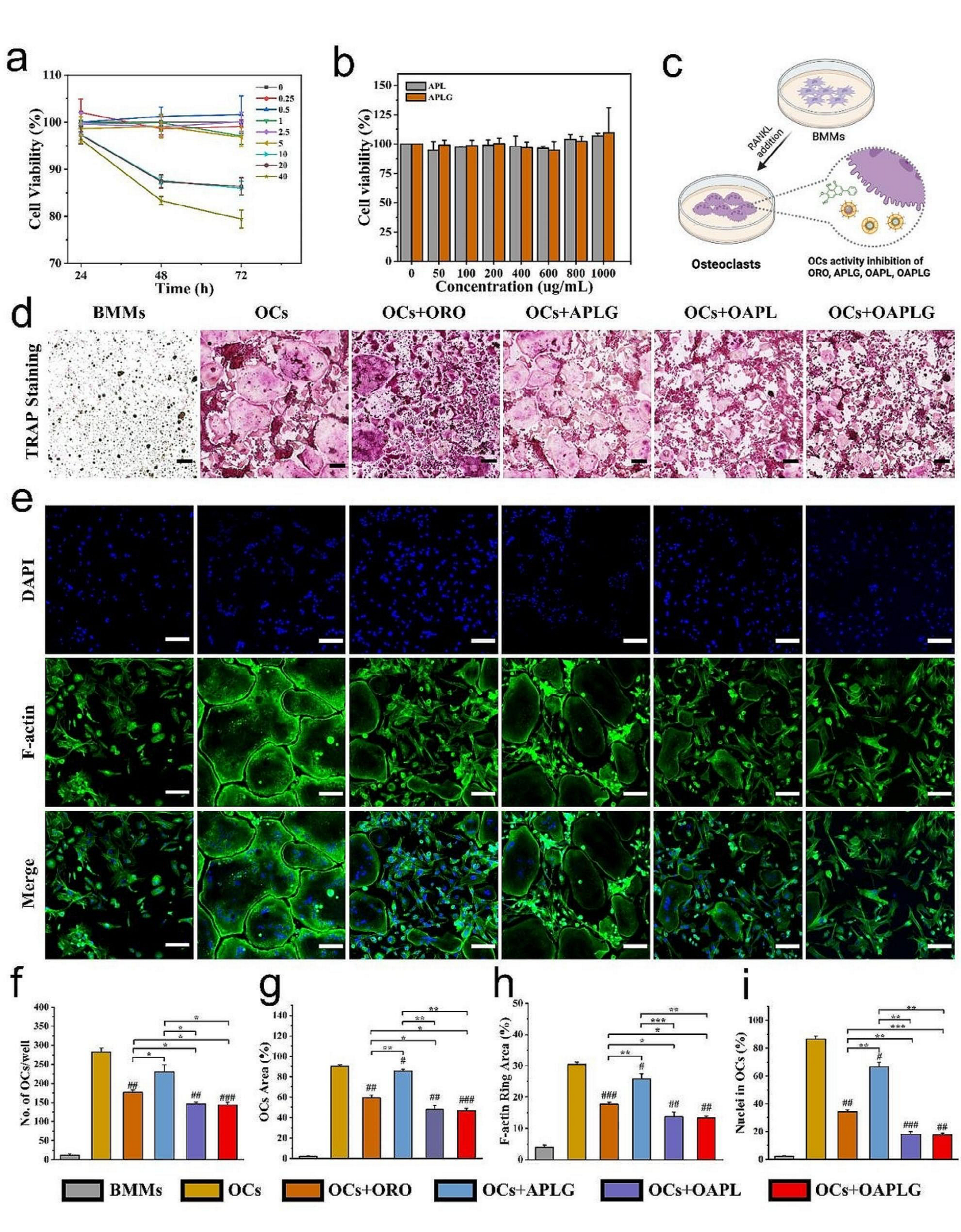
Interfering effects of OAPLG on OCs formation. (**a**) Effect of different concentrations (μM) of ORO over time on BMMs activity. (**b**) Effect of different concentrations of nanocarriers on BMMs activity. (**c**) A scheme illustrating the experiment groups for OCs inhibition. (**d**) TRAP staining of OCs upon indicated treatments. (**e**) F-actin ring (green) and multiple nuclei (blue) and merged images of OCs. (**f**) The number of OCs and (**g**) the percentage of area occupied by OCs, based on the data from Fig. 3d. (**h**) Measurement of the F-actin ring area and (**I**) calculation of the percentage of nuclei within OCs, based on the data from Fig. 3e. Scale bar in all images: 100 μm, *n* = 3. *P < 0.05, **P < 0.01, ***P < 0.001; #*P* < 0.05, ##*P* < 0.01, ###*P* < 0.001 vs. OCs

To explore the inhibitory effect of OAPLG on the maturation and function of OCs, we initially studied the cytotoxic effect of varying concentrations of ORO as well as nanocarriers on BMMs, which serve as osteoclast precursors. The results indicated that ORO concentrations up to 5 μM had a negligible impact on BMMs proliferation over 72 h, whereas concentrations exceeding 5 μM began to diminish cell viability on the second day (Fig. 3a). Consequently, for subsequent experiments, we selected 5 μM as the optimal drug concentration. Moreover, the nanocarrier materials without ORO loading showed no cytotoxicity to BMMs even up to the concentrations of 1000 μg/mL by the CCK-8 assay, proving a good biocompatibility (Fig. 3b). Considering that OAPLG contains ACC as an additional component that is believed to influence the cellular activity of osteoclasts [47], nanocarriers ACC/PL/DGlu6 without drug loading (abbreviated to APLG) was applied as a positive control in experiments related to biological evaluation.

TRAP staining and quantitative analysis were conducted to evaluate osteoclastogenesis. We seeded BMMs in 96-well cell culture plates to adhere, and then underwent different treatments and induced with complete medium containing RANKL and M-CSF for 5 days (Fig. 3c). As shown in Fig. 3d **and f-g**, while all experimental groups (ORO, APLG, OAPL, and OAPLG) show inhibitory effects on BMM differentiation, the OAPL and OAPLG groups exhibit significantly lower quantities and area of TRAP-positive multinucleated osteoclasts compared to the ORO and APLG groups. Moreover, no significant differences were observed between the OAPL and OAPLG groups, suggesting that the bone-targeting peptide DGlu6 had no discernible impact on BMMs activity. Considering the crucial role of podosome F-actin rings in the establishment of acidic zones and the enhancement of bone resorption activity, we employed FITC-labeled phalloidin and DAPI to assess the impact of the experimental groups on the F-actin ring. Confocal imaging and quantitative analysis revealed a significant reduction in both the F-actin ring area and the nuclear proportion following OAPL or OAPLG treatment, whereas a less pronounced reduction was observed after ORO or APLG treatment (Fig. 3e **and h-i**). We have previously reported that ORO binds to LPL and inhibits the formation of filopodia in osteoclast precursors, which are essential actin reorganization structures for cell fusion [34]. The inhibitory effect of APLG wrapped with ACC on osteoclast formation may be explained by the interference of acid neutralization with the sealing structure of osteoclasts and the inhibition of RANKL stimulation on osteoclast precursors [44]. These results underscore a synergistic inhibition of osteoclast formation upon treatment with pH-responsive nanoparticles (OAPL and OAPLG) containing both ORO and ACC.

**Fig. 4:**
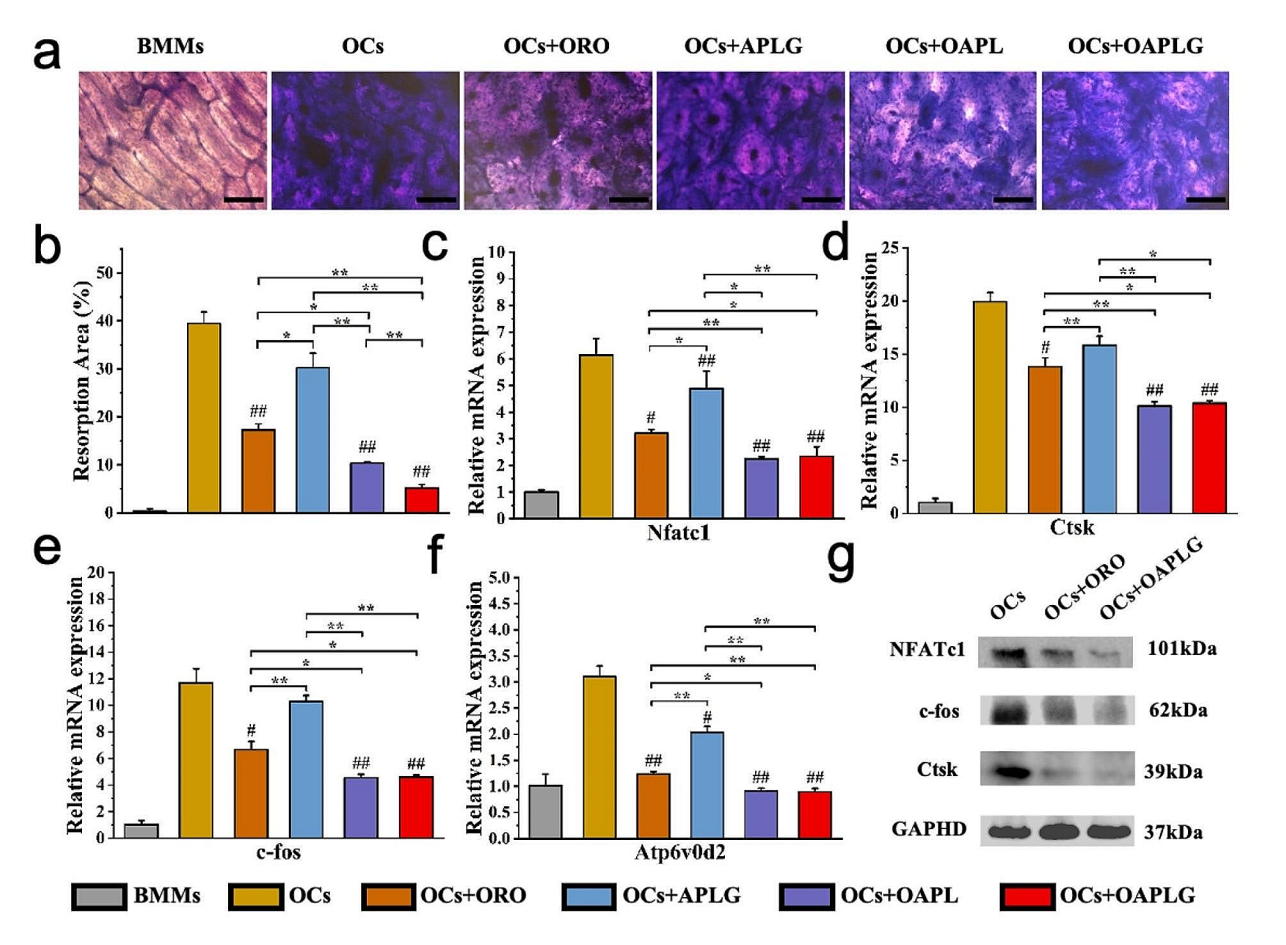
Effect of OAPLG on OCs function and function-specific genes. (**a**) Microscopic images showing areas of OCs-mediated bone resorption after 10 days of specific treatment, scale bar 100 μm. (**b**) The percentage of bone resorption area by Fig. 3a. (**c-f**) Quantitative real-time PCR assessed that OAPLG downregulated expressions of osteoclast function effector genes (**c**) NFATc1, (**d**) Ctsk, (**e**) c-fos, (**f**) Atp6v0d2. (**g**) Western blotting analysis of expressed NFATc1, c-fos and Ctsk. *n* = 3, *P < 0.05, **P < 0.01, ***P < 0.001; #*P* < 0.05, ##*P* < 0.01, ###*P* < 0.001 vs. OCs

We conducted further investigations into the inhibitory effects of OAPLG on osteoclasts using bone slices as a model. Following a 10-day co-culture of osteoclasts and bone slices treated with different experimental groups, all ORO-containing and APLG groups exhibited a reduction in the absorption area compared to the control (Fig. 4a-b). Notably, the OAPLG group demonstrated the most remarkable reduction in the area of bone resorption (depicted as black areas), indicating superior anti-bone resorption effects within the osteoporosis microenvironment. This enhanced effect can be attributed to synergistic therapeutic effects, as well as the presence of bone-binding peptides on the surface of OAPLG. In addition, we conducted qRT-PCR and WB to investigate the molecular-level effects of OAPLG on osteoclastogenesis. These assays confirmed OAPLG’s osteoclast-suppressive properties, acting as both osteoclasts’ inhibitors and acid neutralizers, resulting in a synergistic effect. The expression of osteoclast function effector genes (NFATc1, Ctsk, c-fos, and Atp6v0d2) was downregulated in all experimental groups, with the OAPLG group showing a more significant effect than ORO and APLG (Fig. 4c-f). Corresponding protein production (NFATc1, c-fos, and Ctsk) was also significantly reduced by OAPLG (Fig. 4g). These factors within osteoclasts play pivotal roles in the degradation of organic bone components. Previous studies have demonstrated that ORO can effectively hinder NFATc1 activation, leading to a reduction in the nuclear translocation and transcription levels of NFATc1, which subsequent decrease correlates with a lowered expression level of other osteoclast-specific genes [31]. Additionally, some studies have demonstrated nanoparticles possessing acid-neutralizing properties contribute to the diminished expression of pivotal genes and proteins such as NFATc1, Ctsk, c-fos, and Atp6v0d2 [42]. These studies provide a comprehensive explanation for the aforementioned results. Specifically, pH-responsive OAPLG nanoparticles act as osteoclast inhibitors and acid neutralizers, which have broader implications for cell homeostasis and consequently impact bone resorption.

### Reversal of OVX-induced osteoporosis with OAPLG

**Fig. 5:**
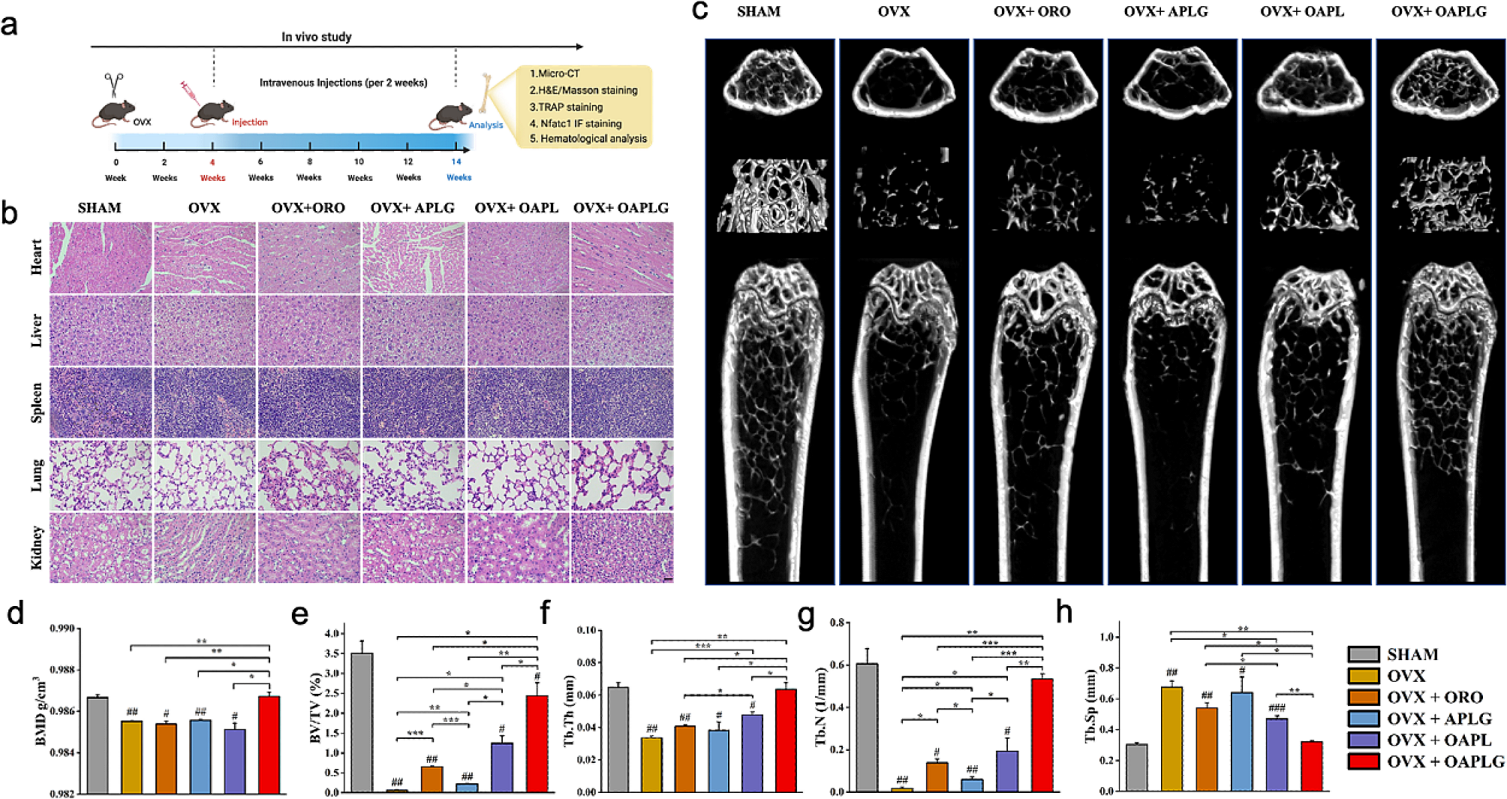
OAPLG effectively reduces bone loss from OVX. (**a**) Schematic illustration of the arrangement of animal experiments to assess the anti-osteoporosis potential of OAPLG. (**b**) H&E staining of major organs, scale bar 100 μm. (**c**) Representative Micro-CT images of the distal femur transverse section, longitudinal section and ROI reconstructed trabecular structure collected from ovariectomized mice treated with ORO, APLG, OAPL, OAPLG and the untreated control group. (**d**) BMD; (**e**) BV/TV; (**f**) Tb. Th; (**g**) Tb. N; (**h**) Tb. Sp. *n* = 3, *P < 0.05, **P < 0.01, ***P < 0.001; #*P* < 0.05, ##*P* < 0.01, ###*P* < 0.001 vs. Sham

To further validate OAPLG’s superior bone protection against osteoporosis in vivo, we employed an ovariectomy (OVX) osteoporosis mouse model for this study. Initially, we conducted a hemolysis assay to evaluate the acute toxicity of OAPLG. Remarkably, even at a high concentration of 300 μM, OAPLG showed negligible effects on mouse red blood cells (RBCs) (Fig. S4a-b). Following the successful establishment of the OVX model, we administered ORO, APLG, OAPL, OAPLG, or a saline solution via tail vein injections every two weeks, as illustrated in Fig. 5a. There were no adverse events or deaths recorded during ovariectomy and subsequent dosing. In addition, none of the mice in the ORO, APLG, OAPL and OAPLG groups showed significant damage to major organs compared to the OVX or sham-operated groups. Histological analysis through H&E staining of major organs in OAPLG-treated mice revealed no abnormalities, confirming the excellent biocompatibility of these nanoparticles (Fig. 5b).

Micro-CT scanning was employed to observe bone loss and microarchitectural changes in mouse bone tissue (Fig. 5c) and to analyze parameters including trabecular BMD (Fig. 5d), BV/TV (Fig. 5e), Tb. Th (Fig. 5f), Tb. N (Fig. 5g), and Tb. Sp (Fig. 5h) at 10 weeks post-treatment. The micro-CT images confirmed substantial trabecular bone loss as a result of OVX, confirming the successful establishment of osteoporosis. Following our in vitro findings, both ORO and the supplementation of targeted ACC (APLG group) demonstrated the alleviation of osteoporosis and an increase in compact bone density compared to the OVX group. Notably, OAPL nanoparticles lacking bone-binding capability also significantly ameliorated osteoporosis compared to OVX, ORO, and APLG groups, indicating a substantial enhancement in microstructure and bone volume. These results can be attributed to the acid-responsive property of OAPL, which enhances ORO accumulation within the acidified extracellular microenvironment of osteoclasts. Prior investigations have unveiled that ORO exhibits the capacity to diminish osteoclast formation and bone resorption through the suppression of osteoclast precursor fusion and the inhibition of NFATc1 activation [31], which was confirmed by the increased bone volume in all ORO-treated groups in our study. In addition, it has been reported that acid-neutralizing nanoparticles can destroy the podosomes of osteoclasts and promote the release of extracellular vesicles containing a large amount of RANK receptors, which can bind to RANKL in serum and further inhibit bone resorption process [42]. Moreover, calcium supplements have been reported to stimulate bone formation by modulating the osteogenic microenvironment [41]. Thereby, the synergistic effects of using ACC could exert further beneficial effects on therapy. As expected, the synergistic effects of ORO/ACC combined with bone-targeting modification (OAPLG group) further enhanced trabecular bone health, ultimately achieving the most favorable microstructure and bone volume in the micro-CT assay. Quantitative analysis of micro-CT scanning, especially BMD and BV/TV, is widely used as predictors for assessing osteoporosis. Consistent with the conclusions mentioned above, femur BMD was significantly different between the SHAM group and the OVX group, indicating the successful establishment of the osteoporotic model. OVX group significantly reduced BMD and BV/TV in mice. Mono-therapy with either bone-targeted ACC (APLG) or ORO effectively reversed this decline. Notably, combination therapies (OAPL and OAPLG) exhibited superior effects. Similar trends were observed in the Tb. Th and Tb. N compared to the OVX group. Furthermore, the OVX group increased Tb. Sp in the treated subjects, which was effectively reversed by OAPLG, outperforming mono-therapy. Importantly, OAPLG consistently outperformed OAPL across all experiments, providing strong evidence of OAPLG’s promising bone-targeting capability and positive effects in osteoporosis therapy.

**Fig. 6:**
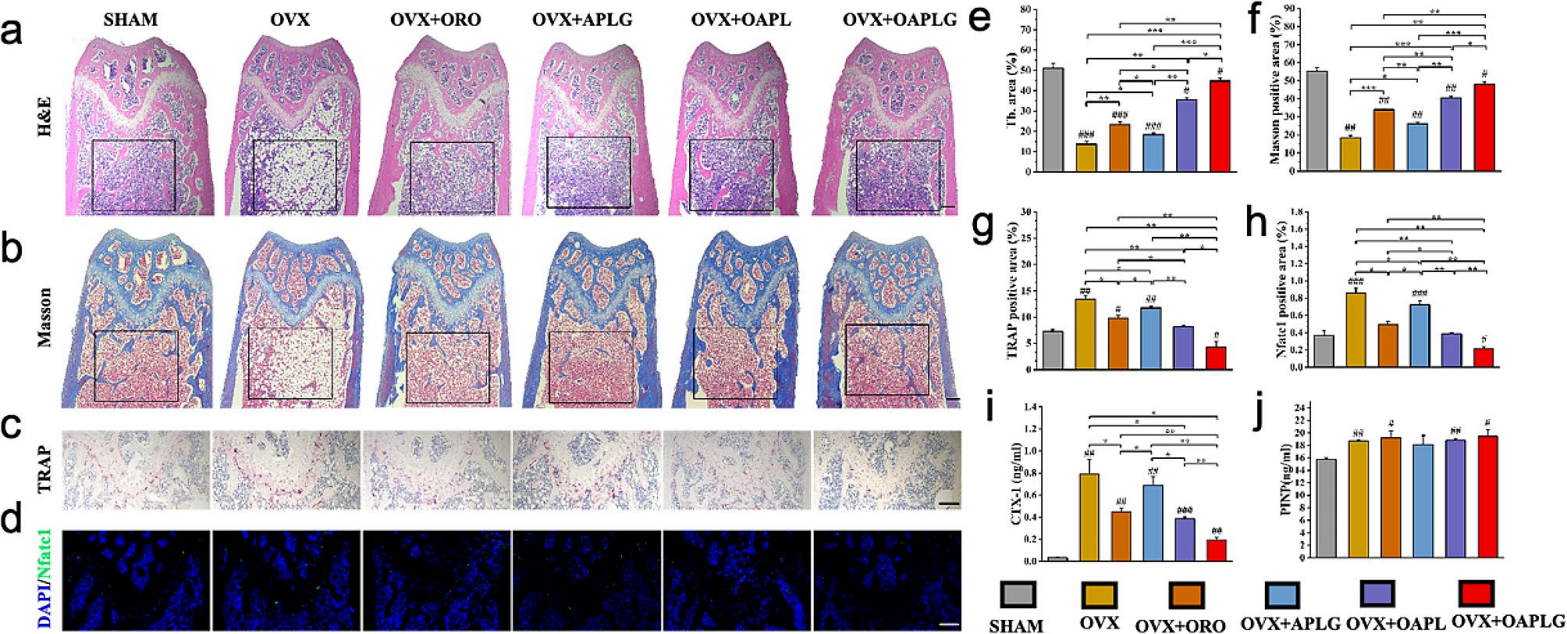
Tissue staining and serum chemical index detection. The distal femurs were subjected to H&E staining (**a**), Masson staining (**b**), TRAP staining (**c**), and Fluorescent immunostaining of NFATc1(**d**), and (**e-h**) are the semi-quantitative results of their important indicators respectively. (**i-j**) are the serum level of CTX-1 and PINP. Scale bar in all images: 200 μm, *n* = 3, *P < 0.05, **P < 0.01, ***P < 0.001; #*P* < 0.05, ##*P* < 0.01, ###*P* < 0.001 vs. Sham

Next, histological assessments through H&E and Masson staining were used to analyze bone volume and collagen content within bone tissue (Fig. 6a-b). The OVX group exhibited a higher presence of adipose tissue compared to the other groups. Additionally, all ORO-containing groups exhibited significant improvements in trabecular interconnectivity compared to OVX. Importantly, the OAPLG group, with the bone-targeting peptide DGlu6, demonstrated significantly enhanced trabecular bone structure compared to the OAPL group and closely resembled the SHAM group in observed effects (Fig. 6e-f). Consistently, TRAP staining demonstrated that OAPLG effectively reduced the presence of TRAP-positive OCs in the trabecular bone, implying that OAPLG significantly prevented osteoclast differentiation and maturation in vivo (Fig. 6c). This observation is supported by the quantification of osteoclast parameters, which demonstrates a reduction in the TRAP-positive area treated with OAPLG groups (Fig. 6g). This result can be explained by previous studies that ORO could inhibit osteoclast formation [34]. Since NFATc1 activation can be suppressed by ORO, we observed lower expression levels of NFATc1 in osteoclasts treated with OAPLG in our in vitro experiments. This was further confirmed by the reduced expression of NFATc1 protein in vivo using fluorescence immunoassay (Fig. 6d **and h**). Additionally, the bone catabolic markers in peripheral blood serum, CTX-1 were significantly lower in OAPLG than those in the OVX, ORO, and APLG group via ELISA analysis (Fig. 6i). The absence of variations in the bone anabolism marker, PINP, implies that OAPLG does not exhibit adverse effects on osteoblasts in vivo (Fig. 6j). These results highlight the pH-responsive and synergistic effect of ORO/ACC combined with D-Glu6 bone targeting in mitigating the impact of OVX-induced changes and promoting bone health. This suggests the potential utility of OAPLG as a promising therapeutic approach in osteoporosis.

## Conclusion

In this study, we developed an acid-responsive, bone-targeted nanoparticle (OAPLG), designed to deliver the lipophilic drug Oroxylin A with responsive release in the acidic microenvironment of osteoclasts. Interestingly, through the synergistic effect of combining drug therapy with acid neutralization therapy, OAPLG effectively impedes the viability, differentiation, and function of OCs, offering a potential treatment for metabolically active osteoporosis. ORO was coated on ACC and wrapped with phospholipids to stabilize nanoparticles and achieve precise release in an acidic microenvironment. The addition of bone-targeting peptide D-Glu6 endowed it with bone-targeting specificity, thus further reducing off-target effects and improving bioavailability. Compared with free ORO treatment, OAPLG showed significant efficacy in reversing bone loss and alleviating microstructural damage in osteoporotic mice, without affecting bone synthesis metabolism. In addition, OAPLG also effectively inhibited the expression of OCs-related functional genes, which serve as therapeutic targets or biomarkers for osteoporosis. This study highlights the great potential of multifunctional nanoplatforms as medical interventions for osteoporosis.

### Electronic supplementary material

Below is the link to the electronic supplementary material.

Supplementary Material 1

## Abbreviations

α-MEM: Alpha-minimum essential medium
ACC: Amorphous calcium carbonate
APLG: ACC/PL/ DGlu6
BMD: Bone mineral density
BMMs: Bone marrow macrophages
BV/TV: Bone volume/ tissue volume
Ctsk: Cathepsin K
CTX-1: C-terminal telopeptide of type I collagen
DAPI: 4’,6-Diamidino-2-phenylindole
DGlu6: D-glutamic acid hexapeptide
DL: Drug loading
DLS: Dynamic light scattering
DOPA: 1,2-dioleoyl-sn-glycero-3-phosphate
DPPC: 1,2-dipalmitoyl-sn-glycero-3-phosphocholine
FBS: Fetal bovine serum
FITC: Fluorescein isothiocyanate
H&E: Hematoxylin and eosin
M-CSF: Macrophage colony-stimulating factor
MALDI-TOF-MS: Matrix-assisted laser desorption ionization time-of-flight mass spectrometry
MW: Molecular weight
MWCO: Molecular weight cut-off
OAPLG: ORO/ ACC/PL/ DGlu6
OCA: ORO coating ACC
OCs: Osteoclasts
ORO: Oroxylin A
OVX: Ovariectomy
PBS: Phosphate-buffered saline
PINP: Procollagen type 1 N-terminal propeptide
PL: Phospholipid
PVDF: Polyvinylidene fluoride
qRT-PCR: Real-time quantitative PCR
RANKL: Receptor activator of nuclear factor-κB ligand
SDS-PAGE: Sodium dodecyl sulfate-polyacrylamide gel electrophoresis
Tb. N: Trabecular number
Tb. Sp: Trabecular separation
Tb. Th: Trabecular thickness
TEM: Transmission electron microscope
TRAP: Tartrate-resistant acid phosphatase
UV-Vis: Ultraviolet-visible spectroscopy
WB: Western blotting
