# Supplementary material for: Smart osteoclasts targeted nanomedicine based on amorphous CaCO3 for effective osteoporosis reversal
Source: J Nanobiotechnology. 2024 Apr 5;22:153. doi: 10.1186/s12951-024-02412-9 (PMC10996086; doi:10.1186/s12951-024-02412-9)
Supplement: Supplementary file 1 — Supplementary Material 1 [file 12951_2024_2412_MOESM1_ESM.docx]

**Supplementary data**

**Smart Osteoclasts Targeted Nanomedicine Based on Amorphous CaCO_3_ for Effective Osteoporosis Reversal**

Biao Yu ^1,2,3,4#^, Qianmin Gao ^1,2,3#^, Shihao Sheng ^5#^, Fengjin Zhou ^6#^, Zhen Geng^1,2^, Yan Wei^1,2^, Hao Zhang^1,2^, Yan Hu^1,2,7^, Sicheng Wang ^1,2,3,8*^, Jianping Huang ^3,4,9*^, Mengmeng Li ^1,2*^, Jiacan Su ^1,2,7*^

^1^ Institute of Translational Medicine, Shanghai University, Shanghai, 200444, China.

^2^ Musculoskeletal Organoid Research Center, Shanghai University, Shanghai, 200444, China.

^3^ School of Medicine, Shanghai University, Shanghai, 200444, China.

^4^ Second Affiliated Hospital, Shanghai University, Wenzhou, 325000, China.

^5^ Department of Orthopedics Trauma, Shanghai Changhai Hospital, Naval Medical University, Shanghai, 200433, China.

^6^ Department of Orthopedics, Honghui Hospital, Xi’an Jiao Tong University, Xi’an 710000, China

^7^ Department of Orthopedics, Xinhua Hospital, Shanghai Jiao Tong University School of Medicine, Shanghai, 200092, China.

^8^ Department of Orthopedics, Shanghai Zhongye Hospital, Shanghai, 200941, China

^9^ Department of Neurology, Wenzhou Central Hospital, Wenzhou, 325000, China.

# These authors contributed equally.

*** Correspondence:**Corresponding Author
w.s.c@sina.com, dr.hjp@163.com, mengmengli@shu.edu.cn, jiacansu@shu.edu.cn

**Table S1.** The primers of genes used in the article.

| **Gene** | **Primer sequence (5‘ to 3’)** |
| --- | --- |
| **Nfatc1-F** | TCCACCCACTTCTGACTTCC |
| **Nfatc1-R** | CTTCGCCCACTGATACGAG |
| **c-fos-F** | GTTCGTGAAACACACCAGGC |
| **c-fos-R** | GGCCTTGACTCACATGCTCT |
| **Ctsk-F** | TCCGCAATCCTTACCGAATA |
| **Ctsk-R** | AACTTGAACACCCACATCCTG |
| **Atp6v0d2-F** | CAAAGCCAGCCTCCTAACTC |
| **Atp6v0d2-R** | GTTGCCATAGTCCGTGGTC |
| **β-Actin-F** | ACAGCAGTTGGTTGGAGCAA |
| **β-Actin-R** | ACGCGACCATCCTCCTCTTA |


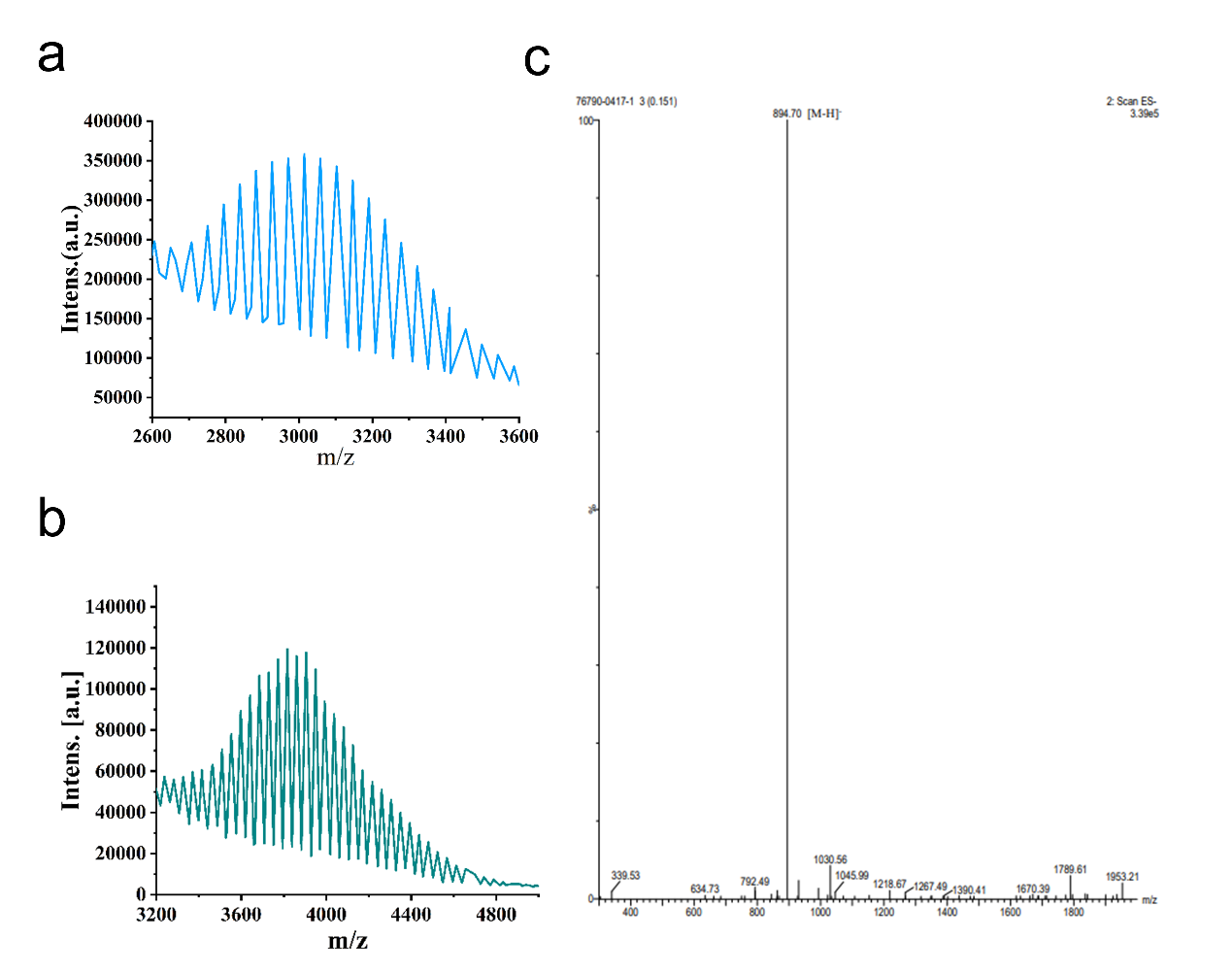


**Fig. S1** The characterization of DSPE-PEG2000-DGlu6. (**a**) MALDI-TOF-MS spectrum detection of the MW of DSPE-PEG-Mal. (**b**) MALDI-TOF-MS spectrum detection of the MW of DSPE-PEG2000-DGlu6. (**c**) MS Analysis of the DCys-DGlu6.


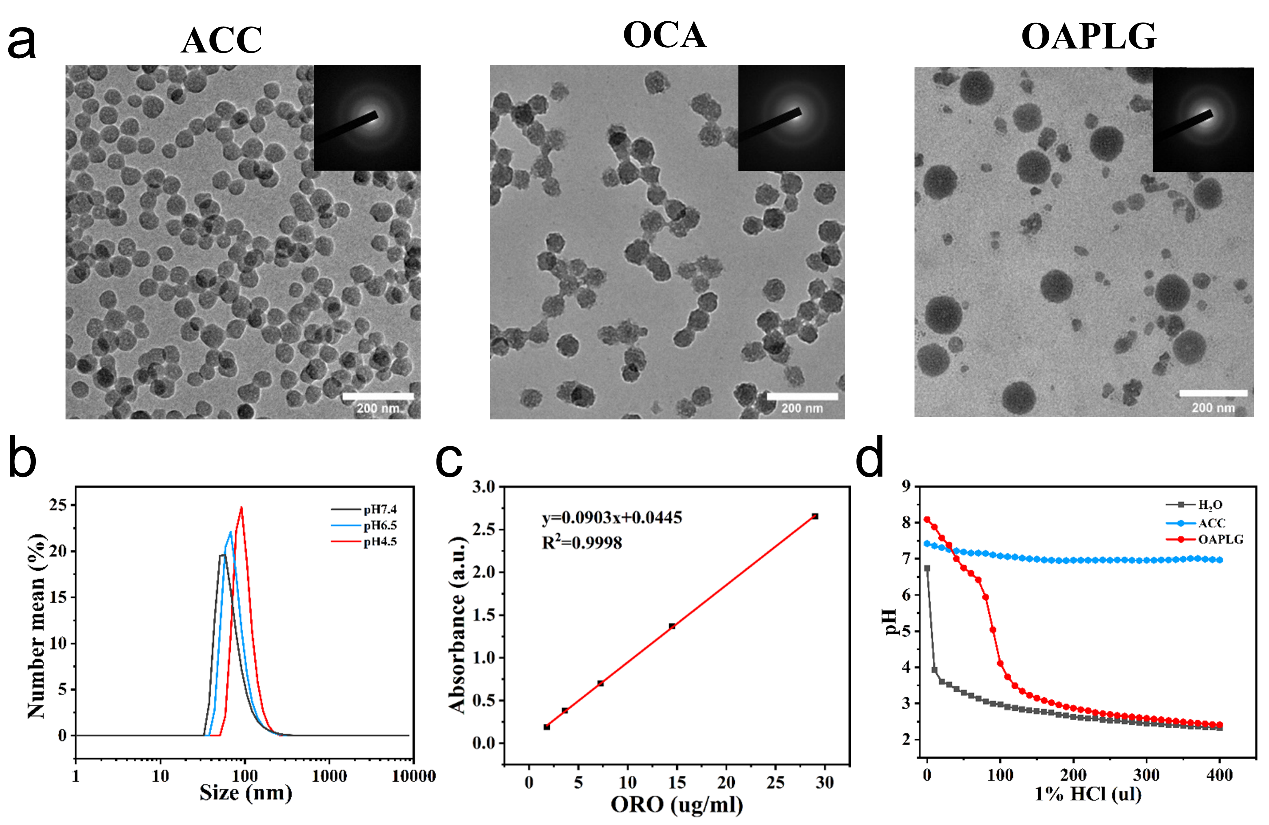


**Fig. S2** Characterization of OAPLG. (**a**) Representative TEM and SEAD images of ACC, OCA and OAPLG. (**b**) Changes in particle size of OAPLG after 4h incubation in different pH buffers. (**c**) The standard curve of ORO by UV-visible spectrophotometer. (**d**) Hydrochloric acid titration experiment.


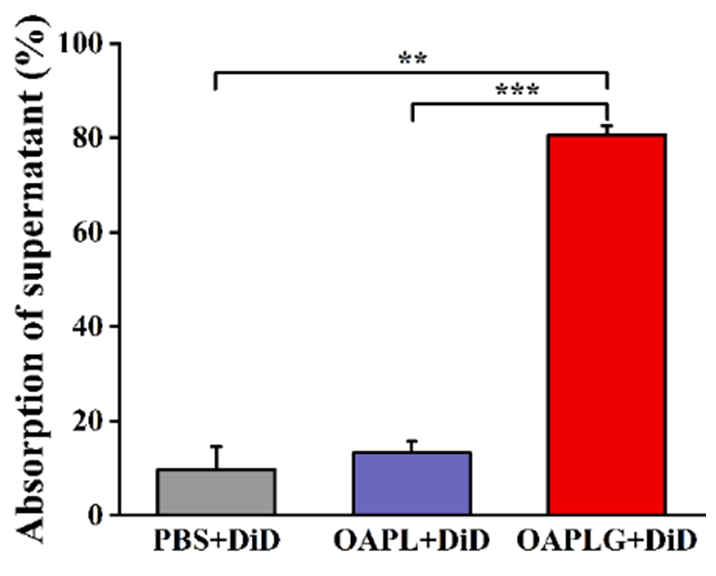


**Fig. S3** The binding capacity of DiD-labeled OAPLG to bone slices in vitro.


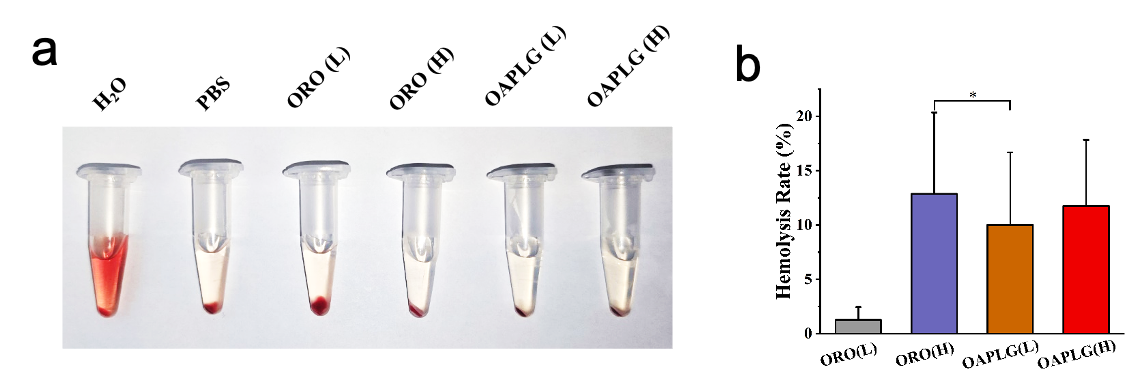


**Fig. S4** Biocompatibility of OAPLG. (**a**) Hemolysis monitoring in OAPLG. (**b**) Hemolysis rates based on Figure S4a.
